# Palliative Care, Intimacy, and Sexual Expression in the Older Adult Residential Care Context: “Living until You Don’t”

**DOI:** 10.3390/ijerph192013080

**Published:** 2022-10-12

**Authors:** Catherine Cook, Mark Henrickson, Vanessa Schouten

**Affiliations:** 1 School of Clinical Sciences, Auckland University of Technology, Auckland 0627, New Zealand; 2 School of Social Work, Massey University, Auckland 0745, New Zealand; 3 School of Humanities, Media and Communication, Massey University, Palmerston North 4442, New Zealand

**Keywords:** person-centred care, social citizenship, social death, sexual expression, aged residential care

## Abstract

Commonly, frail older adults move to residential care, a liminal space that is their home, sometimes a place of death, and a workplace. Residential facilities typically espouse person-centred values, which are variably interpreted. A critical approach to person-centred care that focuses on social citizenship begins to address issues endemic in diminishing opportunities for intimacy in the end-of-life residential context: risk-averse policies; limited education; ageism; and environments designed for staff convenience. A person-centred approach to residents’ expressions of intimacy and sexuality can be supported throughout end-of-life care. The present study utilised a constructionist methodology to investigate meanings associated with intimacy in the palliative and end-of-life care context. There were 77 participants, including residents, family members and staff, from 35 residential facilities. Analysis identified four key themes: care home ethos and intimacy; everyday touch as intimacy; ephemeral intimacy; and intimacy mediated by the built environment. Residents’ expressions of intimacy and sexuality are supported in facilities where clinical leaders provide a role-model for a commitment to social citizenship. Ageism, restrictive policies, care-rationing, functional care, and environmental hindrances contribute to limited intimacy and social death. Clinical leaders have a pivotal role in ensuring person-centred care through policies and practice that support residents’ intimate reciprocity.

## 1. Introduction

Residential care for older adults has evolved in developed countries that have followed the ‘indoor relief’ welfare state model of the United Kingdom, where the 1942 Beveridge Report [1] built on the historical workhouse model to create a ‘cradle to grave’ social security for people of all classes [2]. Residential care is increasingly becoming the place of palliative care and death for older adults. Once people move to residential care this domicile is typically their final home [3]. The New Zealand context has to a large extent adopted the Beveridge model, and more recently added neoliberal embellishments; there is a burgeoning of independent living facilities for relatively well older adults, and progressive levels of care available for people experiencing frailty and the impact of long-term conditions that contribute the degrees of dependency [4]. With increasing care needs, residents have reduced privacy around intimacy and sexual expression as they may require increasing staff assistance around the practicalities and the furnishings required to be physically close to a partner. In the early 21st century, a new norm has evolved for life care outside the family which effectively atomises dependent elders, fostered by a neoliberal approach to health and aged care. A market has been created, and a business case made to fill it [5]. This capitalisation of older persons adds complexity to the construct of person-centred care, with considerations of whether this is a relational model or one in which the resident is positioned as consumer; we consider this point in more detail below. This market-driven model is quite different from the post-war social insurance model originally envisioned by Beveridge: old age has been commodified and transformed into profit-making opportunities by private entities. This neoliberal context has a deleterious impact, with the emphasis on risk-averse policy management rather than person-centred care [4].

People are increasingly frail on entering residential care. In New Zealand 43.7% of people who move to supported residential care live for less than a year, and 24.3% move into care in the final three months of life [6]. Approximately 47% of adults in New Zealand use residential care for late life care, with this increasing to 66% for those 85 years and over [7]. Of note, currently most people in residential care are of European ethnicities (who are 64.1% of the total population), with only 4.7% indigenous Māori and 2.3% of Pacific people using residential care [8,9], although they constitute 16.5% and 9.0%, respectively, of the total population [10]. Although cultural notions of caring for elders within families may account for these low proportions of utilisation in non-European cultural populations, the number of people in care in all groups is predicted to rise significantly in the next decade with a shift away from inter-generational living [8]. Residential has become the de facto hospice regardless of ethnicity for many older adults [11]. For the purposes of this article, palliative care refers to the care of people whose condition is life-limiting, and end-of-life care relates to when people are actively dying [12]. The palliative care context differs in residential care compared to the role of hospice services for younger adults. Internationally the hospice philosophy and funding models are designed to reserve in-patient care for those with complex symptom management issues for stabilization, for respite care and for those who are actively dying whose preference is a hospice death [13]. The hospice model primarily focuses on at-home support throughout the palliative and active dying stages. However, in the residential care context, the key predictive entry factors relate to curtailed independence around frailty and cognitive impairment, rather than disease exacerbation and the imminence of death [14]. This differing reason for entry to residential care has meant that traditionally residential care has not been identified as providing hospice-type care [11]. This under-accentuated role of palliative care in residential care can mean staff are ill-prepared and inadequately supported around end-of life-planning and dialogue with residents and family members [15], impacting on person-centred care.

The discourse of person-centred care is commonplace in the residential care context with abundant literature on the topic. However, there is a dearth of publications that link the concept of person-centred care to palliative care in this situation. The hallmarks of person-centred care include foregrounding the person rather than biomedical diagnoses and symptoms; upholding dignity; the provision of tailored, flexible care; maximising the person’s involvement and control (where possible) of decisions; and recognising the fundamental importance of relationships [16,17]. The uncritical ubiquity of the term person-centred care poses the risk that managerial and care staff believe that by using it they are enacting it and centralising all aspects of the residents’ personhood. The gap between rhetoric and reality is noted in an integrative review by Byrne et al. [18]. These authors identify definitional commonalities in themes of valuing both the care receiver and the care provider. The partnership space integral to person-centred care is compromised in time-poor contexts [18]. However, substantial evidence indicates a person-centred focus is fragmented for reasons beyond care rationing, particularly in relation to residents’ expressions of intimacy and sexuality [19,20,21,22,23], a point we explore shortly.

The concept of person-centred care has a complex, multi-stranded genealogy. In the 1940s the humanistic psychologist Carl Rogers developed a person-centred approach to care that emphasised human potential towards growth and what he termed self-actualisation. A hallmark of the latter is that quality of life is defined by the person. Rogers believed flourishing was possible and relevant into the late stages of life. However, this late life potential required relational conditions of unconditional positive regard and opportunities for closeness and intimacy [24]. The disability rights movement in the 1970s added to the push for care that eschewed biomedical reductionism and instead focused on people’s subjectively defined needs and resources. The movement also argued for societal inclusion and accessibility rather than the responsibility for adaption being placed on people with disabilities [25]. Disabilities were re-interpreted as located in environmental barriers rather than individual abilities. The application of the concept of person-centred care to the lives of older adults was led by Kitwood [26], who emphasised the relational aspects of the approach. O’Dwyer [27] argues that the rise of neo-liberalism has distorted the earlier origins of person-centred care that affirmed relational engagement and instead accentuates a consumer-based model of individualism and choice. We concur with Loughlin [28], who argues that the term is operationalised with ubiquity in health documents without the implementation being clearly conceptualised.

How care is conceptualized is also impacted by managers’ social constructs about care homes. Ettelt et al. [29], in a UK qualitative study with 24 care home managers noted that the ways care managers thought about personalised care differed depending on the overall metaphors they applied to care homes. Their analysis identified three different architectural conceptualisations: institution, home, and hotel. In turn, these viewpoints resulted in divergent ways the care managers made sense of the personalised relationship with residents. While managers wanted to distance their approach from the institutional motif, they concurred that with care rationing and routinisation, aspects of the institution could not be erased. Regulatory compliance around safety and risk at times intruded on personalised care. The family metaphor was preferred by most managers in Ettelt et al.’s study. This orientation included encouraging intimacies between staff and residents that are usually discouraged as boundary breaches in acute care settings, such as hugging and kissing. The notion of care home as hotel led some managers to rename typical home spaces as commercial places: for example, the dining room was described as a restaurant. A hotel metaphor was used in the endeavour to teach staff the quality of care required. We are struck by the irony that staff might imagine they would provide better care in a hotel than in a care home.

We draw from the related concepts of social and sexual citizenship and authentic ageing to inform our understanding of person-centred care. We argue that it is naïve to arrive at an understanding of person-centred care in residential care without identifying major obstacles. A key hindrance is ageism. O’Dwyer [27] identifies that consumerism and modernity/postmodernity result in the cultural rejection of anyone old, particularly people who bear the hallmarks of ageing, including physical frailty and cognitive impairment: this exclusion is exacerbated by a neoliberal understanding of the commodification of persons and the value of their economic productivity. Although there is veneration of older people, for example in Confucian-based and most indigenous cultures, the COVID-19 pandemic has highlighted openly ageist discourses internationally, see for example [30]. The concept of social citizenship is largely predicated on individual agency to self-govern. ‘Social citizenship’ is a term coined by Marshall [31], a sociologist in the United Kingdom, who argued that ideally citizenship entails access to social, civil, and political rights. Brannelly [32] considers that the ability to be self-governing is a narrow and dehumanising view that robs persons of dignity and inclusion. An inclusive alternative conceptualisation of citizenship is “one who cohabits with others” [32] (p. 120), with the emphasis on interdependence, support, and reciprocity. This reconceptualization is also termed relational citizenship [33,34]. Sexual citizenship affirms enfranchisement and belonging, including people’s need for affection and intimacy that transcends frailty and cognitive impairment [35]. The concept of authentic ageing, rather than the narrower biomedical construct of successful ageing, is proposed by Hughes [36] as intersecting with that of citizenship. Authenticity in this context relates to holding both the potential for growth and change until death and the acknowledgement of the inevitable vulnerabilities that come with the finality of life, or as one of our participants put it, “living until you don’t”.

As researchers and practitioners, we consider that the construct of person-centred care is socio-political in nature and vulnerable to reductionist distortion. Optimally, person-centred care in the residential care setting enhances social and sexual citizenship and communitas, understood as a shared community of experience that includes residents, families, and staff [37]. Communitas is associated with the development of connection and intimacy in the coming together of people experiencing liminality. We consider that residential care is an exemplar of this betwixt-and-between space, both a home of sorts and a workplace [38,39]. Authentic ageing as integral to person-centred care means that all parties—staff, families, and residents—view residents as people with ongoing connections to families and communities, with the possibility of change and growth as well as people with embodied vulnerabilities that create interdependence and dependence. Person-centred care informed by relational citizenship includes seeing residents as having the capacity for intimacy, sexual expression and closeness that is not necessarily commensurate with cognitive ability [35].

Several decades of literature, and legal cases that have made international headlines, highlight that the bounds of person-centred care in the residential palliative care context stop short of consideration about residents’ needs for affection, intimacy, and sexual expression [19,20,21]. Isolating individuals from affectionate or intimate touch (even intimate touch that is paid for) runs contrary to the notion of citizenship and person-centred care. Institutional or managerial anxiety about putative risk—or the appearance of risk—either to a resident or institutional reputation, means that in many cases risk management or risk avoidance is prioritised over full citizenship of residents and their wellbeing or flourishing. In this way residents are further vulnerabilised. Although there are pockets of excellence internationally, it is commonplace for a gulf between policies and practice to exist, and between aspiration and the necessary leadership and staff education to fully implement those policies [22,23]. More broadly New Zealand is a democracy with relatively liberal laws and social policies including marriage equality for gender and sexually diverse people and the decriminalization of homosexuality and prostitution [23]. Aged care regulations support rights around sexual orientation and expression [40] but policy implementation is fragmented [41]. When people in New Zealand enter residential care, they are required to have appointed an Enduring Power of Attorney, usually a family member, who will take over decisions on their behalf should the resident lose cognitive capacity [42]. However, paternalistic custom and practice and limited staff education in residential care mean that family preferences over-ride residents’ choices, even when residents have capacity [38]. Families are unnecessarily consulted about matters about which they have no legal right or need to know, and residents’ rights to privacy are ignored.

Frail older adults in care are vulnerablised by the attitudes and anxieties of staff, families, and other residents. This vulnerability is made worse where cis-heteronormative assumptions mean that gender and sexually diverse residents fear or directly experience disenfranchisement, stigma, and discrimination [23,43], or feel forced into covert identities. The built environment additionally creates obstacles unless residential care homes are designed with intimacy and privacy rights in mind [44]. Residential care is an ambiguous space in relation to privacy, due to the constructed communal context of people who are not organically part of the same community or tribe (being both home and workplace) and because residents’ care needs mean regular intrusions on public/private boundaries [45,46]. Organisational culture makes a significant difference to residents’ quality of life, and that culture reflects what is valued by leadership; how communication occurs; how rules and procedures shape care; and what counts as success [15,46]. Leadership and role-modelling are especially significant in upholding relational citizenship around intimacy and sexual expression [22,47].

The research question guiding the larger project asked how are ethical decisions being made about expressions of intimacy and sexuality in aged residential care? [41]. The broader project focused on examining how everyday decisions are made, which result in tacit practice ethics. The study impetus came from clinical and legal practitioners seeking clarity about the ethics and complexities of consent that occur commonly in residential care. Despite these requests, throughout the larger research project, we also met opposition to our study from some senior physicians and the leadership of a national advocacy organisation for older adults. The position taken in these instances has been that there are much more compelling issues the sector is facing and that our research focus on intimacy was a nice-to-have extra-the ‘icing on the cake’ of residential care. We consider that a person-centred care approach that is inclusive of social and sexual citizenship is fundamental to ensuring life worth living rather than the social death and warehousing of frail older adults [48]. The aim of this article is to consider how a person-centred approach to residents’ intimacy and sexual expression can be supported by families and staff throughout palliative and end-of-life care in the residential care context. The results highlight that person-centred care discourses and practices must be inclusive of a social citizenship conceptualisation of people with the potential for and right to intimate and sexual expression through their entire lives. This person-centred orientation is inherently political, focused on enfranchisement and equity. This position requires managers and clinical leaders to engage with at times complex ethical situations, and not to resort to an over-reaching duty of care argument that excessively vulnerabilizes older adults [48].

## 2. Materials and Methods

These qualitative data are drawn from a national two-arm mixed method study in Aotearoa New Zealand. Thorne’s [49] methodological approach, interpretive description, was used. This methodology draws from factual material and social constructionist analysis to aid investigation of the ‘messy’ world of healthcare. The social constructionist paradigm focuses on how meanings are created, sustained, negotiated, and interrupted [50].

### 2.1. Procedure and Participants

Purposive sampling was used to recruit a national sample of staff, residents, and residents’ family members from large (>100 beds), medium (50–100 beds), and small care providers (<50 beds) to stratify the sample selection for the qualitative arm of the study. Staff completed a survey and interviews were conducted with staff, residents, and family members. The research team provided an introductory presentation about the study for staff at each facility. Senior staff then provided fliers to residents and family members and posters were visible in the facilities. Residents either contacted the team directly or via a family or staff member. Semi-structured interviews were conducted between October 2018 and October 2019 (concluding before COVID-19 lockdowns) with participants recruited from 35 residential care homes across the country. Project staff conducted 61 interviews with 77 participants recruited from the participating facilities. Interviews were conducted at a time convenient for the participants, in a safe, uninterrupted venue in the facility. Interviews were completed as follows: staff, 36 interviews, residents, 26 interviews with 28 people (couples interviewed together); family members, 12 interviews with 13 people. This article focuses primarily on staff interviews although a full report that includes all interviews is available at https://mro.massey.ac.nz/handle/10179/15720 (accessed on 10 July 2022).

### 2.2. Ethical Considerations

The study was approved by a University Human Ethics Committee (NOR18/25). Participation in the study was voluntary. Participants were informed about the study; respect for confidentiality and anonymity were discussed, and written consent was obtained before interviews. The ethics committee was satisfied that the research team had demonstrated expertise in sexuality research.

### 2.3. Data Analysis

Audio recorded interviews were transcribed. Thematic analysis, guided by Braun and Clarke’s [50] six steps of data analysis, was undertaken to identify key themes. These steps involve the following: initial data familiarisation; assignment of preliminary codes; search for patterns across the dataset; clustering themes; naming themes; report writing. To ensure rigour with inter-rater reliability, the research team independently read and coded all transcripts [51]. All team members then reviewed others’ coding and through meeting and dialogue collectively developed themes. Analysis was both deductive and inductive and it was only during the data analysis phase that it became evident to the research team that the palliative and end-of-life context of residential care was entwined with staff, family and residents’ accounts about intimacy and sexual expression and that this point is not foregrounded in the extant literature. Interview questions brought forth this connection. Questions to residents included: Given that varying degrees of acquaintances, friendships and more intimate relationships are a part of most people’s adult lives, how easy or difficult is it to continue with these relationships here? In your opinion, what is the role of staff in managing and supporting people’s personal choices and safety in relation to intimacy and sexuality? Questions to family members included: Did the transition to RAC separate your relative from important networks, relationships, friendships, intimate partners? What intimate relationship issues would be of concern for you? Questions for staff included: To what extent do you think that fostering connections, including supporting intimate relationships, is the role of staff? What, if any benefits do you consider might occur through staff, structures and policies allowing for people to continue intimate relationships in residential care? Can you give some examples from your experience?

## 3. Results

The following themes were identified through the analysis process: care home ethos and intimacy; everyday touch as intimacy; ephemeral intimacy; and intimacy mediated by the built environment. Together these themes highlight the particularities of intimacy and sexual expression during the palliative and end-of-life phases in the residential care context. Residents were reliant on staff values as to whether they are treated as socially alive and therefore support for intimate expression was considered part of person-centred care. Participant accounts indicated that when staff treated residents as socially alive, staff friendliness and connection through everyday touch became a meaningful part of residents’ world. Other residents at times provided a level of intimacy that staff and families either encouraged or thwarted. The built environment typically presented obstacles to intimacy and privacy, with design features focused on surveillance and the assumption that bedrooms would have a single occupant.

### 3.1. Care Home Ethos and Intimacy

The values and advocacy of clinical staff appeared to impact on whether supporting intimacy and sexual expression was considered part of care. Sexual citizenship was contested:

*There’s some staff who just can’t tolerate it [any expression of intimacy], it’s the way they feel personally, and you have to say to them, “Hey, you can’t say that they can’t sit together and hold hands and stroke each other, because they’ve got that right, and they need that, and if they’re happy in the moment, and it’s relieving that loss and that sadness and that misery, and even if it’s half an hour of happiness. Well, just because you can’t stand it, and you think, ‘because that’s not their husband’, how can you say no to that?”*

*(Staff 8)*

This staff member worked in a care area for people living with dementia and highlighted that a person-centred approach to residents expressing intimacy was effectively shaped by caregivers’ personal moral positions. This paternalism created occasions where staff automatically assumed it was their right (or obligation) to intervene in intimate expressions by residents, and this was noted by other participants:

*Over the last 25 years maybe now I’ve noticed a shift towards husband and wives. They’re a little bit more accessible; they can share a room. I still think there’s horror surrounding maybe sexual activity, but certainly if a relationship develops between residents within the rest home, of a non-platonic nature, or if one’s come in and the spouse is outside, or it’s a partner [who] is outside, I still think we’re quite obstructive, because we don’t have the places for them to go. Their rooms are small, and I’ve just found that my colleagues’ attitudes around relationships is that there’s a cut-off age, and that’s usually probably 65, and then after 65, whether it be handholding, kissing, or anything; just should stop, and that it’s, it just doesn’t happen.*

*(Staff 7)*

This participant highlighted the interplay between ageism, staff interactions and the assumptions that have informed the design of the built environment. Each of these features appear to reinforce the other to the point that the staff participant has noted a change over several decades. Staff also noted that in the palliative care context, the involvement of caregivers may be required to support the practicalities of physical intimacy between a couple. A staff member described facilitating a couple’s intimacy through discussion with the resident and a plan:

*What he wanted is to have a cuddle. How do we facilitate that? What he said to me is that “I can’t really transfer from the chair to bed.” So, I wanted them to be private, so I did the ‘do not disturb’ sign and everything. So, I said, “Okay, what you need to do is we’ll talk to your nurse here, talk to the other nurse there [where his partner was], and then when you go over there, when she’s available, you can always ask the nurse for you to be transferred into her bed, and from there that’s it.”*

*(Staff 10)*

Some staff perceived that they were part of the intimate lives of residents, providing emotional and physical comfort, even though they may be the only staff person facilitating such care:

*We spend our lives as adults growing up to look for that all night cuddle with someone special. And isn’t it the cuddle, that intimacy of cuddle that is the most comforting thing? I think that’s something that people in residential care get robbed of very much, is that they don’t get any of that touch, they don’t get any lovely touch. They don’t get hugged every day unless I’m there.*

*(Staff 1)*

One staff participant related a story about upholding sexual citizenship around a resident’s preferences with her lingerie; she had to contest the family’s view that the resident was socially dead:

*When you are faced with a decision, think first and foremost if this would be my mother, assuming you absolutely adore and love your mother, what would I do? Real story: you look in the [resident’s] drawer of underwear and they’re all ripped and ugly. This person has most probably another month to live and loves underwear and loves real nice underclothes. Well, the nurse comes to us and says, “What can we do? This is ugly. Can the family buy them something?” The family goes, “No.” Then you can be totally assured we will [make this purchase]. We have actually encountered the family response of, “No, there’s no point,” [because the person is dying] and, we’re going, “Oh my god, there’s a whole point.”*

*(Staff 3)*

A manager described the ways his personal views had strong links to his practice, including advocating for and facilitating sexual citizenship for palliative residents:

*Like, my own values; for me it’s like I’m living each day as it is my last. For this particular resident, if tomorrow he died or she died, I know that she had a good day, even having a massage, or sex with a sex worker, isn’t that amazing that we have been part of that, that I advocated and facilitated that; she died happily.*

*(Staff 10)*

Despite New Zealand’s legal protection of the rights of gender and sexually diverse people, the interviews highlighted participant perceptions that residential care was not yet necessarily a safe space for these residents. A daughter commented:

*As long as gay people stay away from me I don’t care what people do; that’s other people’s lives…. The family has got to be happy about it as well, and every family is different. It might not be a drama for some families. My sister and I would be quite horrified if that [partnering with a woman] happened to mum.*

*(Family 7)*

Another family member commented about a resident who was vocal about any signs of affection between residents:

*It would be like if there were a couple of gay gentlemen in there; she would be absolutely horrified, she’d be mortified, this woman, and it would just be a massive ‘no’ for her. So, I would imagine she would make that very difficult for those people.*

*(Family 8)*

Another family member spoke about her brother, who was dying of cancer in a Catholic residential care facility:

*I don’t know how you could [be intimate] in a place like that. His being gay would maybe be a problem for him; it’s not a problem for me. Maybe the home, I don’t know what their attitude would be. I don’t know whether they know he’s gay or not. Maybe they don’t. He’s had a wife. He’s got children, so he doesn’t present as a gay man.*

*(Family 12)*

This comment highlights the uncertainty family members and residents encounter when facilities do not clearly signal support for gender and sexually diverse people. However, a few staff participants spoke of their facilities readying for a shift at an organisational and policy level by going through the national Silver Rainbow training programme provided by a non-governmental organisation. This programme provides facilities with an organisational needs analysis and education programme. The following participant was not aware of any current gender and sexually diverse residents but affirmed the importance of readiness for change:

*We are certainly prepared for a gearing up to a change probably sooner rather than later. So yes, we have our Silver Rainbow tick, and we have worked quite hard on a lot of policies and procedures and that side of things.*

*(Staff 16)*

While this preparation is significant, the participant did not appear to appreciate that change has already happened, in that gender and sexually diverse people already live in residential care, hidden in plain sight.

A staff member drew attention to the liminal space that is residential care, purportedly a home and yet without the hallmarks of home, in part because of such large groups of otherwise unconnected people being brought together:

*So, we can make it home-like, but it is actually not a home. It is still an institution, and there are rules, and even in your own house; I mean, I can’t just do what I want. I live with somebody else, and there’s rules; we’ve got house rules. So, we’re quite often told here, “This is their home, and they should be allowed to do what they want, and blah-blah-blah.” Yes, I get that, but yes also they’re living with 46 other people, and I only live with one other person, and I can’t do whatever I want, and she can’t do whatever she wants, because there’s rules.*

*(Staff 7)*

This participant expected new residents to fit in with institutional rules, rather than being a part of determining the parameters of communal living. However, another staff participant emphasised that in the facility where she worked there were significant efforts to create a home-like space where intimate others could feel welcome:

*We have a 24-hour a day open door, seven days a week. So, from a cultural perspective, we’ve got a Māori lady and her daughter stays [overnight] at times. We either provide a room or they can stay in the room. This room here has been turned into a whānau [extended family] room where they can stay if there’s three, four or more, and a shower is available. We serve breakfast here.*

*(Staff 3)*

The manager described his efforts to navigate this public/private space and to privilege the concept of home:

*“[A resident said] All I wanted is for you to call me by name, not darling, not love; call me by my name, because that’s the only thing I have, and then respect my privacy,” and that’s what we’re trying to implement. That’s what we’ve been telling [staff] all the time; reminding them [staff] that [even though] this is a hospital…that this is their home. We’re invading their home, so all we need to ask is consent to get into the room or whatever.*

*(Staff 10)*

It is clear in the above quote that the contested context of residential care impacts on the possibility of person-centred care. The family member of a resident who had recently died commented on her grief at the attitudes of other residents towards any intimate expression between her and her husband:

*There is very little chance for couples to enjoy any sort of sexual relationship. Even holding hands is sort of sniggered at by everybody in the room. Most of the rooms have got single chairs with arm rests in between and you can’t sit close. You can’t sit and have a cuddle, and if you do people are laughing at you. Neither of us realised how much this would impact on our lives as a loving couple. We had always valued our private intimate moments together, but now every moment had to be spent in public with others watching our every touch. Even facial expressions of love were seen by others and probably commented on.*

*(Widow family member 1)*

The communal context of residential care along with the limitations of the built environment, discussed further below, detract from the possibility of person-centred care around intimacy and sexual expression. Resident participants also spoke of not even knowing if they were allowed to be sexually intimate in the residential care context. In our study we found limited evidence of facility staff proactively educating and guiding residents with respect to intimacy. This silence communicated that intimacy was an unspeakable topic. The result was that residents did not voice their concerns to staff, and assumed that intimacy was not permissible. One resident recounted declining his wife’s intimate advances because he was unsure if it was ethical to respond:

*My wife suffers from dementia. As far as I’m concerned, to the best of my knowledge, sexual relationships with me are not on. I don’t know whether… I mean, some little while ago I woke up with [wife] by my bed crying. I asked her why she was crying, and she said, “I thought you were dead. You were cold and I hadn’t seen you for four days.” Then she got all gooey, if that’s the right word, which I did not respond to, as I possibly should have done, because I don’t think that a person with dementia is in a position to make those sort of decisions. The following day, when I tried to raise the question with her, she said, “What’s it all about?” and she’d forgotten all about it. So, if I had taken advantage of her, to put it that way, that would have been very wrong I think. I don’t think that she’s in a position to make those decisions.*

*(Resident 12)*

The resident’s experience highlights the often-complex decisions older adults are grappling with alone, even in established relationships. The interviews highlighted that the care home ethos is shaped by all members of the community-staff, residents and families.

### 3.2. Everyday Touch as Intimacy

Some staff highlighted in effect that person-centred care is a relational practice where the boundaries of friendliness and professionalism overlap to offer residents meaningful engagement:

*So, that when you are touching people intimately and you’re cleansing someone, you can still touch them warmly and do that very consciously. Well, there is still that boundary there and you’ve still got to be professional, but it doesn’t have to lack authenticity or warmth.*

*(Staff 1)*

Personalising care requires taking intimate notice of residents:

*I think that most of us here all get a cuddle, or an arm around them, or, “You’re looking gorgeous today,” you know, give them a kiss or, “I better get you a new lippy, [lipstick]” or something like that. I think that a lot of them in care miss out on a physical touch and I think that’s important. It can just be an arm around you saying, “Hey, you need a haircut,” or whatever, but I think that’s important that they have that touch of another person that actually cares. I think that’s very important.*

*(Staff 23)*

A family member spoke of seeing what she termed a “craving” for touch when she visited her relative in a dementia care unit:

*There were people who were very tactile with us; like when we visited there were particular older women who would want to come and give you a hug or sit next to you and pat your hand. Physical touch was something they were obviously really craving. They would always want to come and give the kids [visiting children] a hug*

*(Family member 5)*

This same family member spoke of touch as a human right:

*I just think it seems like a fundamental human right really, to have that physical touch and have the ability to continue that part of your existence, even though you might have changed where you’re living, or how you’re living. I do understand that the issue of consent can become pretty fraught in an environment where people aren’t necessarily always clear on their behaviour and why they’re doing the things they’re doing, so I can understand that it’s really difficult. It’s a conundrum for where the boundaries are with that.*

*(Family 5)*

This family member in effect appeared to indicate that prioritising residents’ access to touch should not automatically be over-ruled because consent issues can be complex. Participants’ views highlighted that individual staff acted in effect as ‘champions,’ affirming their relational role with residents and the importance of touch.

### 3.3. Ephemeral Intimacy

Part of citizenship enfranchisement in person-centred care involves staff and family members being aware of residents’ capacity for new significant relationships to develop, with the concomitant grief when these relationships end through the death of one of the parties. A family member noted the loss for her mother, and that these losses were compounded by the segregated nature of the care facility where most of her companions were likely to die soon:

*They got to know each other a wee bit. [Resident] got sick and died, as they tend to do, and that’s one of the things, as far as mental health and wellbeing goes, I noticed with my mother, is that she finds that she gets to know people, but then they die as they tend to do at a certain age, and in a place like that, you’re surrounded by very elderly and often very unwell people. People are going to pass on. That’s something which mum has mentioned a few times as being a bit sort of depressing really. Apart from the staff, there isn’t a lot of young life or energy.*

*(Daughter family member 11)*

One daughter showed awareness of her mother as socially alive with the capacity for meaningful connection, albeit fleeting:

*Not long after mum came there was a lady came here; she was 99. She turned 100 in July and mum just adored her; and [Resident] adored her as well. [Resident] would just sit in her chair. She could feed herself. But they had this amazing relationship. It was so lovely. Mum just kind of took care of her. [Resident] died probably a month ago. They had a really strong bond. It was lovely to see.*

*(Daughter family member 2)*

A resident living with Huntington’s disease spoke of the rich intimacy and pleasure a short-lived relationship with another resident brought to her life:

*We fell in love. We had cups of tea and had a lovely time and then he died. We had a sign that we put on the door…. I didn’t want him to die, I loved him. I had him as my friend and for sex, it was good. We used to go to each other’s rooms…. the relationship warmed my heart.*

*(Resident 17)*

These exemplar quotes illustrate the importance of staff noticing and supporting residents’ preferences around companionship and facilitating the development of connections between people. A staff member spoke about their conceptualisation of care:

*I think it is part of our role [fostering intimacy and connection] given that they have come into care and intimacy is part of the care. You are having the whole person come in so you should be catering to all their needs, not just showering, getting dressed. The intimacy and the touch thing is part of who they are as well, so we should be facilitating and fostering that.*

*(Staff 5)*

These participants were clear that what counts as person-centred care is not generic and needs to be adapted to the care context. A staff member indicated that she and her colleagues were stymied in their efforts to enable a couple who had met in the facility to stay together until they died. They were over-ridden by family wishes and appeared not to have any guiding protocols to manage such situations:

*My main thing with it was how are we, as an organisation for aged care, supposed to say, if the families don’t want them to be together, but they want to be together? Because our point of contact is the family-whatever the family says we do. Sometimes it’s just ridiculous….he ended up passing away in the toilet. Then about two days later she passed away. They were both fully for each other, and then the families told us to stop it. We did it. We stopped it. Then he passed away about a week later after we stopped it, then she passed away two days after him. It was just stupid…. It just happened so fast. We didn’t know what to do, as the workers, because we knew that we were kind of taking away their reason for living.*

*(Staff 17)*

In the residential care context, a person-centred approach that includes sexual citizenship may not be possible without family agreement. The staff prioritisation of residents’ physical safety, without thought of innovative solutions was highlighted by a participant who described staff not allowing another resident to lie in bed with him:

*One day [she] was motoring around on her electric scooter and I didn’t know her from a bar soap. As soon as we started talking we felt [participant cries]… we felt we’d known each other for years. She goes in and out of periods when one day she’ll know who I am, and the next day gone [due to dementia]. So I have to be very patient and wait for her to go through her stupidity with the Parkinson’s part of her. Well, I’m not allowed to lie with her because I haven’t got a crash pad that she can fall onto, and that sort of nonsense. But I would have thought that they would understand that I would be catching her every moment and not let her get out into a situation where she could be in danger herself. It’s that kind of interference.*

*(Resident 21)*

Although the above example possibly includes complexities around consent, what we noted in our wider study was typically an overriding notion of a duty of care applied by staff meant it was uncommon for staff to prioritise intimacy and to work actively with residents’ physical and cognitive challenges to find solutions.

### 3.4. Intimacy Mediated by the Built Environment

An ethos of person-centred care is not commonly reflected in the built environment, where architectural design and furnishings reflect a wider assumption that the resident will be single and solitary, and easily accessible to staff. A participant spoke about the obstacles to intimacy throughout her husband’s stay and during his dying hours:

*The rooms were very small. You were lucky if you could get one chair in the room apart from the bed. I would go in there and I would get a chair in his room, and I would sit on the chair. He would say to me, “Where are you going to sleep? How about you sleep in the bed and I’ll sleep on the chair.” He used to worry about me all the time, when he was the one that was sick…. I had only been gone ten minutes and he just died. The only reason I went home was because my legs were swollen up so much from sitting there all day, from six o’clock in the morning till nine o’clock at night. I just couldn’t stand any more and I just had to go home. If it had been possible and I could have crawled onto the bed and lay beside him, so that my legs weren’t hurting, I could have stayed there right till the end, and I would have.*

*(Widowed family member 1)*

A staff member commented that hearing about our research had prompted their staff to reconsider this structural impediment to intimacy:

*So, now we’ve got a quote for a companion bed to put into a room so that we can create that space. Especially if someone is dying, but not necessarily even then, but you can actually jump on the bed and it’s going to be big enough to have a cuddle. You can sleep beside them. We normally set up a Lay-Z-Boy beside a bed at the moment, but obviously comfort wise it’s not quite the same, and you don’t get that full body press; you don’t get that spoon. Isn’t spooning the best thing in the whole wide world?*

*(Staff 1)*

Here we see that staff can be responsive to education and that engagement with our research project had encouraged reflection and brought about a small change. In their comments about spooning, the staff member indicated that they were able to perceive the resident as socially alive, with the capacity to be comforted and experience joy through touch. Staff reported that it was uncommon for double rooms to be available, and that it was a routine practice for couples to be separated to different areas of the facility as individual care needs changed.

*We did have a husband and wife in the dementia wing; he was in a room down one end, and she was down the hall a way, and that was hard because she was wanting to care for him, but his health was very poor, and he eventually died. He’d actually broken a hip and had to go to hospital… then he went to our hospital, and we had to take her down there, and she wanted to stay with him all day, and it became quite a difficult thing. So, in the end the family said, “We can’t come up all the time.” I think they paid someone to take her down there for a while, but that even got too much because she wanted to be there all day. In the end she had to just have that whenever they said that you can go, and she’d cry quite a bit. very hard, because they were devoted.*

*(Staff 8)*

The staff member appeared to accept the status quo of the architectural and care arrangements that created obstacles to intimacy for this couple, and that these aspects were part of the inevitable griefs and losses at the end-of-life to be worked around. From our interviews it appeared that design features reinforced staff and family members’ resignation about the curtailment of intimacy.

## 4. Discussion

Our study findings highlight that key areas that impact on intimacy and end-of-life care include staff and family values; residents’ internalised assumptions of limited rights; the invisibility of policy; and the implicit signals from the architecture and design about whether residential care is a (private) home or a (quasi-public) stepped-down medical facility. This article makes a novel contribution in foregrounding the context of palliative and end-of-life care in relation to intimacy and sexual expression in residential care, which the extant literature does not do. Our results indicate that person-centred care can be only partially realised when residents are not treated as entirely whole persons, including their intimate relationships and sexual selves, and where residents are unaware of their rights; not provided with information and dialogue they need to make decisions; when these decisions are made based on others’ values and judgments; or when institutional risk avoidance or management is prioritised. Our findings add to the literature in demonstrating that such an approach to care matters even and especially into the final days and hours of life.

Our findings indicate that the ethos of a care facility is very important and that families and residents as well as staff contribute to this ethos. To count as person-centred palliative and end-of-life care in the residential care context, carers must acknowledge intimate expression for residents as fundamental to personhood. Our findings show that staff need education and leadership guidance to ensure they are not deferring to family preferences excessively. We concur with Morrissey Stahl et al. [11] that cultural myths about older people’s declining intimacy needs and capacities, along with a narrow cis-heteronormative conceptualisation of sexual expression, mean that the subject is typically ignored by health providers. As evidenced in our study, important connections were either sustained with staff and family support, or truncated in an ad hoc manner, with staff and family preferences and values predominantly shaping care. Importantly, our findings show that staff, residents and family members typically followed custom and practice and personal values rather than any mention of legislation and policy guiding practice.

Although policies and legislation per se do not automatically achieve this goal of sexual citizenship, our findings indicate that education in these matters may contribute to staff readiness for change. Of note, our findings indicated that staff and family participants who witnessed residents’ intimacy losses preferred that arguments about consent capacity did not nullify residents’ apparent longing for touch and connection. This point about the importance of considering wellbeing as having merit as well as consent arguments has been made in the theoretical literature [18,47], and this article adds empirical data where participants voiced this preference. In the theoretical literature, Cook et al. [47] noted common ethical themes in their analysis of residential care intimacy and sexuality policies available internationally. Well-being was a significant consideration, particularly if the resident had some degree of cognitive impairment and usual consent assessments were impacted. Wilkins [52] recommends an individualised case-by-case assessment as this diagnosis does not equate with global incapacity. The medicalisation of sexual consent assessment as if it has equivalence with consent for medical procedures is problematic [53,54]. Additionally, Tarzia et al. [53] contend that evidence of well-being and the right to sexual expression in the residential care context should not be overridden by complex theoretical arguments about capacity. Staff anxiety and lack of education about sexual expression can readily result in excessive social control rather than judicious implementation of a duty of care [54]. This family control was evident in the presented interview data, particularly in the example of where staff felt unable to act on behalf of two residents that families wanted separated despite their death being imminent. In instances where people are living with a dementia.

Our findings highlight that due to the absence of assessment practices and dialogue with residents about intimacy and sexuality, fundamental pieces of information were missing, and impacted on palliative and end-of-life care. An individualised approach to dementia care has been termed narrative care, whereby attention to paid to the overall ‘story’ of the person’s life rather than the imposition of abstract pre-ordained ethical rules [54,55]. Our findings show that the sexual story of residents’ lives is commonly absent from so-called individualized care. Alongside narrative approaches and duty of care, Cook et al.’s [47] policy analysis also noted recurring themes of respect (including respect for diversity), dignity, and human rights. Our findings demonstrate that these points are particularly significant in the palliative care of older gender and sexually diverse adults. There is scant literature about this topic and of note our interviews showed that other residents and family members may contribute to the felt sense of alienation for residents whose lives do not conform to heteronormative views. Henrickson et al. [23,55] noted that managers and staff were pivotal in setting the tone of a facility and how accommodating a facility would be for sexually diverse residents. They also demonstrated a clear generational effect in staff attitudes, with current attitudes of older staff shaped by prevailing attitudes of their formative years. Stevens and Abrahm [56] identify that additional education is important to provide culturally competent care to gender and sexually diverse people in the palliative context. Examples of such care include staff engaging affirmatively with the person’s chosen family, using preferred pronouns, support to maintain preferred gender presentation and meticulous confidentiality [57,58]. Rosa et al. [58] (p. 3) note that heteronormative and cisnormative lenses alienate intimate partners and caregivers and emphasise that “[c]are that is dignified, person-centered, and concordant with a patient’s values must honor the sacred bond of family-however they define it.”

The present study highlights that facility staff preferably exercise a high degree of caution around claims of already realised person-centred care provision in the palliative and end-of-life context in residential care settings, and instead consider such care a dynamic work in progress. We concur with Loughlin [28] that assumptions that this care vision has been implemented already curtail critique of what is likely to be a fragmented application of person-centred care. The personhood of frail older adults who may have some cognitive impairment is contested, and this loss of personhood–social death— is most evident around intimacy and sexual expression [34,35,38].

When we began this study, we were unaware of the extent to which the built environment and related decor would come to shape our view of how strongly architecture and furnishings are linked to recognising residents’ sexual citizenship, including throughout the end-of-life period. The current study is novel in drawing attention to this point outside of the traditional hospice context. We concur with Bellamy et al.’s critique of hospice design [59] (e2), that “architectural homogenization” has contributed to care spaces that emphasise the clinical workplace over home-like environments that provide for the possibility of intimate companionship. Bellamy et al. also note a paucity of architectural terms of reference for creating person-centred palliative care spaces. It is notable how little progress has been made on the architecture of indoor relief and historic workhouse models of care. In novel findings, our participants provided stark examples of how the limitations of the built environment, the furnishing, and attitudes of staff and families in combination meant that some residents experienced restrictions on their desire for intimacy. Zadeh et al. [60] note a breadth of evidence identifying that palliative care design features have real effects, such as frequency and length of visitor stays. Conversely, appropriate furnishings and amenities impact positively on the palliative experience. In the residential care context significant people in the resident’s life are also likely to be older and experiencing frailty and other health issues that warrant consideration. According to findings in our research, palliative design is an integral consideration when endeavouring to provide care that acknowledges sexual citizenship.

The operationalisation of person-centred care is impacted by the way the built environment is interpreted. Our data demonstrate that a liminal sense of place, somewhere between a workplace and a home, mean that palliative sexual expression is contested, and surveillance and institutional convenience may prevail over person-centredness. In relation to liminality, a family member is quoted in the findings referring to the facility as “a place like that.” It was notable across our dataset that family members and residents repeated used this phrase or the alternative, “in a place like this”, rather than ever naming the facility as their home. Although Ettelt et al. [29] do not use the concept of liminality, they usefully point to the impact of care home managers’ architectural conceptualisations of facilities as an institution, home or hotel. Our data highlighted the importance staff and family members placed on facilities being home-like or not and in part made this assessment based on staff expressions of closeness and friendly touch with residents as part of personalised care.

The current study’s findings add to the literature about the importance of staff assuming the role of a type of surrogate family member for residents as part of everyday intimacy in residential care. We consider that O’Dwyer [27] poses a significant question about whether person-centred care positions the person as a relational being or a commodity within a neoliberal, largely privatized residential care market [4]. In our study, staff and family members valued relational care. Chamberlain et al. [61] identified the significant role of staff for residents who were unbefriended and incapacitated. In their study they noted staff spoke of navigating a ‘fine line’ between possible assumptions by other residents of favouritism and providing additional relational care, but nevertheless perceived they had a role as surrogate family. This positioning was also identified by our participants. Recent studies highlight this vital surrogate role staff have played during the COVID-19 pandemic around relational connection and end-of-life care [62,63].

We concur with Theurer et al. [45] that staff need to cultivate opportunities for social connection in residential care that are meaningful for residents, rather than merely providing light time-filling social activities with goals of entertainment and distraction. In short, if residential care takes account of intimacy and the sexuality and sexual life of a resident in an unanxious way, it is likely to provide meaning and purpose for every aspect of that resident’s life.

Like all studies, the data on which we have based our findings, discussion and recommendations have limitations. These are set out in detail at in our full research report, at the link we have included above. While we initially set out to develop a probabilistic sample of facilities stratified by bed number and location, practical and real-world challenges (staff busy with other demands, the mass shooting at the Christchurch mosque, significant flooding requiring evacuation in a rural region) meant that we had to abandon our probabilistic strategy, although we still recruited a stratified quota sample of facilities. Therefore, the facilities (and participants interviewed from these facilities) must be regarded as a sample of convenience, and subject to the usual limitations of such samples. Our participants were richly diverse and reflected multiple points of view. Still, we are likely to have attracted participation both from facilities (through their managers), and from staff, residents and family members who were interested in and comfortable talking with us about issues related to intimacy and sexuality. The ages of residents and the cultural backgrounds of all participants meant that not all possible participants were comfortable with such conversations. Staff were surprisingly eager to talk about these issues since they had encountered them in their daily encounters with residents. Because the purpose of qualitative research is to explore an issue in-depth rather than a population, we make no claims about the generalizability of our findings. Nevertheless, we believe the data provide a useful foundation for analysis and recommendations.

Our recommendations are as follows. If care is to be person-centred then it must be tailored to those receiving care, rather than policy that foregrounds risk management and an institutionally centred approach. Person-centred care means treating residents as socially alive and possibly re-defining what professionalism means, since staff often act as surrogate family for people living in care facilities. Staff, families and residents will benefit from education about legislation, including the purpose and limits of Enduring Power of Attorney to ensure that there is not paternalistic over-reach in families and staff intruding on residents’ privacy and decision-making. Our study highlighted that creating the opportunity for dialogue can enhance staff’ critical reflection, resulting in change. Staff education around the incorporation of the routine assessment of intimacy and sexual expression and gender and sexual identity can shift these topics away from being taboo. While our findings support the utility in facilities having accessible intimacy and sexuality policies that are part of staff and resident induction, it is also apparent that senior staff who champion such documents are fundamental to change. Through role-modelling practice translation they are better positioned to uphold sexual citizenship through end-of-life care [46,64].

## 5. Conclusions

This article highlights the importance of critiquing the ubiquitous use of the concept of person-centred care and considers what aspects of personhood are typically excluded from this care aspiration. We are very mindful of the increased pressures on residential care staff in a post-COVID-19 environment. We believe the reflective, person-centred care approach will result in less stress on staff, as person-centred care increasingly transfers decision-making about social citizenship, wellbeing, and intimacy to the resident. We have recommended a critical approach that draws from the concepts of social and sexual citizenship and authentic ageing to ensure that taken-for-granted ageist and cis-heteronormative assumptions are not driving care decisions. We have highlighted the liminal space that is residential care, and how conceptualisations of institution, home and hotel contribute to care decisions and the identities attributed to residents. The study indicates the vital role of staff, who if willing to do so become an important part of residents’ intimate world. Our participants and our discussion draw attention to the dearth of collaboration between care providers, residents, researchers, architects, and designers, resulting in built environments that defy relational efforts by staff and families to engage in person-centred care. Residential care for older adults is predominantly a palliative and end-of-life care service and it is essential that this care is not the ‘poor relation’ of hospice services provided to younger people. Currently neoliberal, profit-driven drivers eclipse person-centred care [4]. If older persons in care are truly to live—and live fully, in all aspects of their lives—until they die, it will be essential that contemporary residential care and its amenities move beyond historical workhouse models, into lively places of innovation and care for whole persons.

## Data Availability

Not applicable.
